# Chemical Composition and Biological Activity of *Allium cepa* L. and *Allium × cornutum* (Clementi ex Visiani 1842) Methanolic Extracts

**DOI:** 10.3390/molecules22030448

**Published:** 2017-03-11

**Authors:** Željana Fredotović, Matilda Šprung, Barbara Soldo, Ivica Ljubenkov, Irena Budić-Leto, Tea Bilušić, Vedrana Čikeš-Čulić, Jasna Puizina

**Affiliations:** 1 Department of Biology, Faculty of Science, University of Split, R. Boškovića 33, 21000 Split, Croatia; zfredotov@pmfst.hr; 2 Department of Chemistry, Faculty of Science, University of Split, R. Boškovića 33, 21000 Split, Croatia; msprung@pmfst.hr (M.Š.); barbara@pmfst.hr (B.S.); iljubenk@pmfst.hr (I.L.); 3 Institute for Adriatic Crops and Karst Reclamation, Put Duilova 11, 21000 Split, Croatia; irena.budic-leto@krs.hr; 4 Department for Food technology and Biotechnology, Faculty of Chemistry and Technology, University of Split, R. Boškovića 35, 21000 Split, Croatia; tea@ktf-split.hr; 5 Department of Medical Chemistry and Biochemistry, School of Medicine, University of Split, Šoltanska 2, 21000 Split, Croatia; vedrana.cikes.culic@mefst.hr

**Keywords:** onions, *Allium* × *cornutum*, *Allium cepa*, phenolic compounds, antioxidant activity, genotoxicity, antimutagenic activity, antiproliferative activity

## Abstract

Here, we report a comparative study of the phytochemical profile and the biological activity of two onion extracts, namely *Allium cepa* L. and *Allium* × *cornutum* (Clementi ex Visiani 1842), members of the family Amaryllidaceae. The identification of flavonoids and anthocyanins, and their individual quantities, was determined by high-performance liquid chromatography (HPLC). The potency of both extracts to scavenge free radicals was determined by the DPPH (2,2′-diphenyl-1-picrylhydrazyl) radical-scavenging activity and oxygen radical absorbance capacity (ORAC) methods. The DNA protective role was further tested by the single-cell gel electrophoresis (COMET) assay and by Fenton’s reagent causing double-strand breaks on the closed circular high copy pUC19 plasmid isolated from *Escherichia coli*. In the presence of both extracts, a significant decrease in DNA damage was observed, which indicates a protective role of *Allium cepa* and *Allium* × *cornutum* on DNA strand breaks. Additionally, cytotoxicity was tested on glioblastoma and breast cancer cell lines. The results showed that both extracts had antiproliferative effects, but the most prominent decrease in cellular growth was observed in glioblastoma cells.

## 1. Introduction

Oxidative stress is the disturbance between the production of reactive oxygen species (ROS) and antioxidant defenses. Overproduction of ROS can cause damage to important biomolecules, such as DNA, proteins, lipids, and carbohydrates, resulting in a variety of diseases [1]. For this reason, living cells have developed antioxidant systems to control free radicals and lipid peroxidation, and to maintain the oxidative-antioxidative balance [2]. Dietary antioxidants also play an important role in the suppression of oxidative stress, which may cause initiation and progression of various diseases, including cancer. Therefore, the consumption of dietary antioxidants is considered to be an effective tool in preventing diseases that may be caused by oxidative stresses [3]. The genus *Allium* covers more than 750 species distributed all over the northern hemisphere [4]. The members of this genus are known not only as flavored vegetables and spices, but also as medical plants that have been used in traditional medicines [5,6]. Among other *Allium* species, *Allium cepa* L. (the common onion) is one of the oldest plants cultivated worldwide [7]. Many epidemiological studies confirmed that dietary consumption of onions is associated with a reduced risk of developing many forms of cancer and cardiovascular and neurodegenerative diseases [8,9,10]. Their beneficial effect on health is attributed to high contents of biologically active phytomolecules, such as phenolic compounds, especially flavonoids, and several organosulfur compounds [11]. The most abundant flavonoids found in onions are quercetins, namely quercetin-4′-monoglucoside and quercetin-3,4′-diglucoside, which account for more than 85% of the total flavonoid content [12,13]. In addition to flavonoids, onions, especially the red varieties, are a rich source of anthocyanins. The most frequently reported anthocyanins in red onions are cyanindin derivatives, although minor amounts of peonidin, petunidin, and delphidin derivatives have also been identified [14]. *A. × cornutum* (Clementi ex Visiani 1842) is a triploid hybrid onion (2*n* = 3*x* = 24) originating from three parental species (*A. cepa*, *A. pskemense* B. Fedt., and *A. roylei* Stearn), traditionally cultivated in coastal Croatia under the name ‘Ljutika’ (shallot) [15]. Due to its tasty bulbs and leaves, this onion is widely used as a spice and its cultivation has spread to other parts of the world, such as Southeast Asia and Europe. To our knowledge, the chemical composition and biological activity of *A. × cornutum* have not been studied so far. This motivated us to identify and quantify major phenolic compounds present in the bulbs of *A. × cornutum* and *A. cepa* and to assess the biological activity (free radical scavenging potential, antimutagenic activity, antiproliferative activity on cancer cells, and antigenotoxic activity) of these widely used plants.

## 2. Results and Discussion

### 2.1. HPLC Analysis of Flavonols and Anthocyanin and Total Phenolic Content of the Two Onion Species

**Figure 1:**
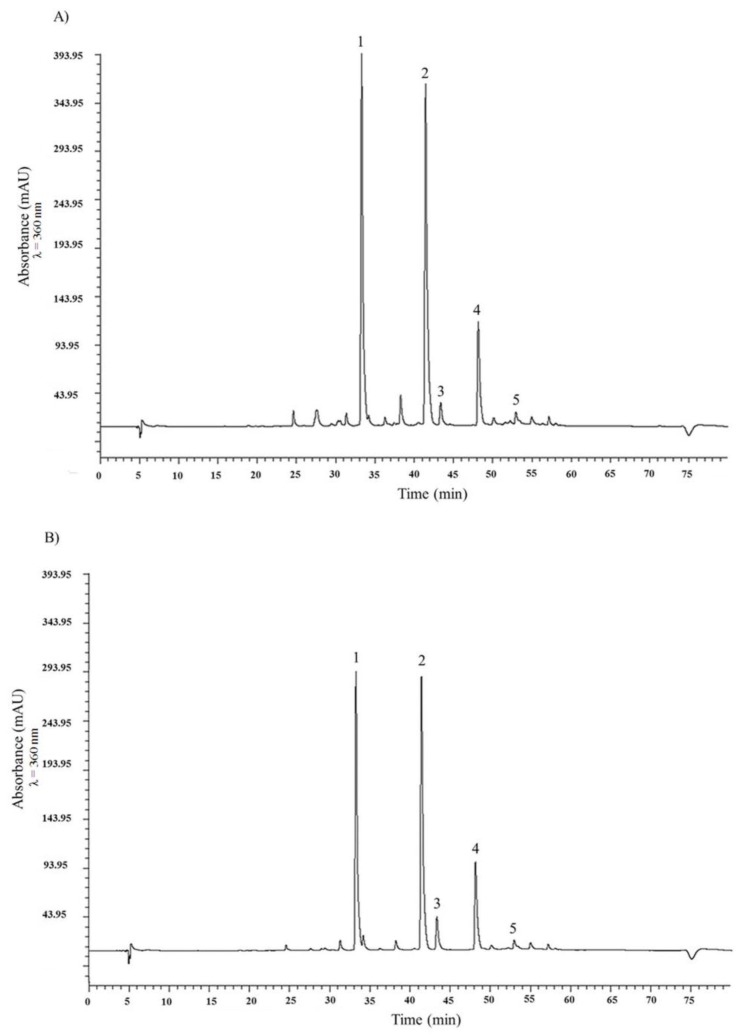
High-performance liquid chromatography (HPLC) chromatograms of *A. × cornutum* (**A**) and *A. cepa* (**B**) methanolic extracts at 360 nm. Depicted are peaks: (**1**) quercetin 3,4′-diglucoside; (**2**) quercetin 4′-monoglucoside; (**3**) quercetin; (**4**) ishorhamnetin; and (**5**) kaempferol.

Two major and three minor fine peaks were successfully resolved by high-performance liquid chromatography (HPLC) UV-VIS analysis of flavonols in *A. × cornutum* and *A. cepa* methanolic extracts (Figure 1). The resulting chromatograms were qualitatively similar, which is in line with other reported studies [16,17,18,19,20]. Here, the two major peaks were identified as quercetin 3,4′-diglucoside (**1**) (retention time (*t*_R_) for *A. cepa* 33.26 min; *t*_R_ for *A. × cornutum* 33.30 min) and quercetin 4′-monoglucoside (**2**) (*t*_R_ for *A. cepa* 41.41 min; *t*_R_ for *A. × cornutum* 41.45 min). The three minor peaks were identified as myricetin (**3**) (*t*_R_ for *A. cepa* 43.34 min; *t*_R_ for *A. × cornutum* 43.37 min), quercetin aglycone (**4**) (*t*_R_ for *A. cepa* 48.13 min; *t*_R_ for *A. × cornutum* 48.17 min), and isorhamnetin (**5**) (*t*_R_ for *A. cepa* 52.94 min; *t*_R_ for *A. × cornutum* 52.97 min). Two main quercetin conjugates, (**1**) and (**2**), together account for about 80% of the total flavonol content in both onions (Table 1).

Similar concentrations of the two quercetin conjugates were previously observed in other studies [16,17,18,19], confirming that the two are indeed predominant flavonols in onions. However, in this work, somewhat higher concentrations of (**1**) and (**2**) were observed in *A. × cornutum*, which agrees with the higher total phenolic content (TPC) (*A. × cornutum* TPC 6.63 ± 0.31 mg caffeic acid (CA)/g dry weight (DW); *A. cepa* TPC 6.24 ± 0.23 CA/g DW) accounted for in this onion species.

Our obtained TPC values show a good correlation to those reported by Santas et al. [21]. In their study, TPC values of the two methanolic extracts of Spanish onion varieties ranged from 5.15 to 6.33 mg gallic acid equivalents (GAE)/g DW for white onions, and 2.48 to 2.58 mg GAE/g DW for Calçot de Valls onions. However, it should be noted that TPC values could vary depending on the location of the cultivar and on the solvent used during the extraction process [1,21,22,23,24].

Furthermore, quantification of individual anthocyanins was carried out using HPLC (Table 1) where three anthocyanins in *A. cepa* and four in *A. × cornutum* were identified. Three anthocyanins, equal in both onions, were further identified as peonidin glucoside (**6**), petunidin glucoside (**7**), and malvidin glucoside (**8**). Interestingly, the fourth anthocyanin, delphidin glucoside (**9**), was found only in the *A. × cornutum* extract (Figure S2A,B). The only study reporting the presence of delphidin glucoside in Tropea red onions was reported by Gennaro et al. [14]. In addition to delphidin glucoside, they have also identified petunidin derivatives, and together these accounted for ~30% of the total anthocyanins in the onion bulbs.

### 2.2. Antioxidant Activity

In order to determine the antioxidant potential of our extracts, two tests were performed, 2,2′-diphenyl-1-picrylhydrazyl (DPPH) and the oxygen radical absorbance capacity (ORAC) assay. Both onions showed mutually similar DPPH and ORAC values at a concentration of 100 μg/mL (Table 2). However, slightly higher antioxidant activity was observed for *A. × cornutum* in the ORAC assay, which can be attributed to a slightly higher phenolic content. The high antioxidant scavenging activity of *Allium* species was reported in other studies where it was shown that this property depends on the existence of both phenolic and organosulfur compounds [24,25,26,27,28,29].

### 2.3. DNA Nicking Assay

Taking into account that both onions have a relatively high amount of phenolic compounds, we wanted to further investigate their potential in protecting DNA from ROS. In that sense, the pUC19 plasmid that was exposed to Fenton’s reagent served as a biological probe.

**Figure 2:**
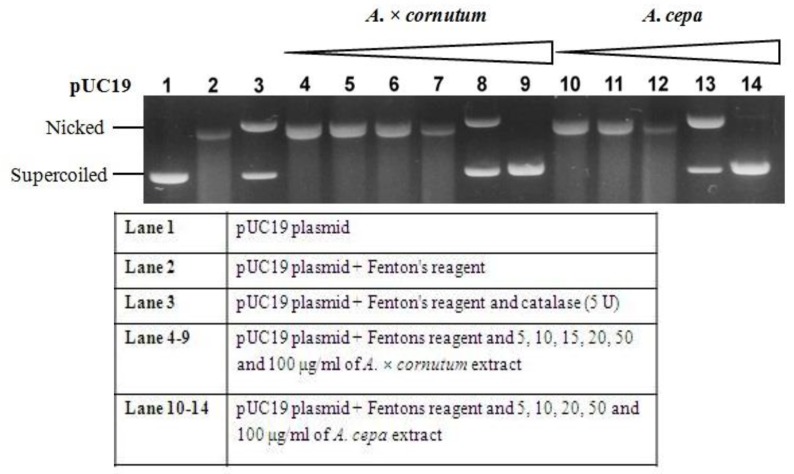
Protective effects of both onion extracts on supercoiled pUC19 DNA.

Hydroxyl radicals generated by the Fenton reaction cause the oxidatively induced DNA strand breaks to yield their open circular or relaxed form [1]. Figure 2 shows the DNA-protecting effects of *A. cepa* and *A. × cornutum*, where it can be seen that lower extract concentrations correlate to poor DNA protection and an increase in open circular and linear forms of DNA. On the contrary, higher concentrations show significant free radical scavenging activity and the ability to maintain a supercoiled form of DNA. Our results clearly indicate that the preservation of an intact circular plasmid is concentration-dependent (5–100 µg/mL).

Since both extracts have a relatively high amount of phenolic compounds (quercetin and its derivatives) which act as antioxidants, we presume that they are responsible for promoting good scavenging activity. Several other previous studies have, in turn, also reported that high phenolic content prevents DNA damage caused by hydroxyl radicals [1,30].

The antioxidant activity of phenolic compounds is associated with the total number and position of the hydroxyl functional groups which are able to reduce highly oxidizing free radicals such as superoxides, peroxyls, aloxyls, and hydroxyls [31]. Other than ROS reduction by hydrogen donation, antioxidant action can include the suppression of ROS formation by chelating metal ions, scavenging ROS and inhibiting oxidases [6]. Since phenolic compounds act as antioxidants, it is justified to conclude that a higher phenolic content leads to a stronger antioxidant capacity. This conclusion agrees with our findings which also showed that TPC values are in correlation with DPPH scavenging and ORAC values for both onions.

### 2.4. Comet Assay

**Figure 3:**
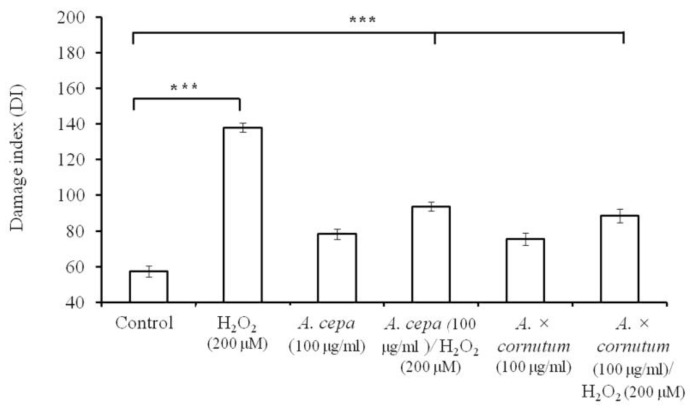
DNA damage index (DI) of treated human leukocytes: control, H_2_O_2_ (200 μM), *A. cepa* (100 μg/mL), *A. cepa* with H_2_O_2_, *A. × cornutum* (100 μg/mL), and *A. × cornutum* with H_2_O_2_; *** *p* < 0.001.

The comet assay was performed to determine the possible protective effects of both onions on DNA damage in human leukocytes (white blood cells; WBCs). Incubation of leukocytes with the DNA damage-inducing reagent (H_2_O_2_, 200 μM) resulted in a significant increase of DNA damage compared to the untreated control (*** *p* < 0.001) (Figure 3).

Cells treated with 100 μg/mL *A. × cornutum* or *A. cepa* extracts showed only a moderate level of DNA damage, indicating that the presence of phytochemicals in onions can also provoke oxidative DNA damage. This is considered to be a structural property of phenolic compounds which can act as both antioxidants and prooxidants at the same time. In the presence of oxygen, some transition ionic metals, such as Cu^2+^ and Fe^2+^ or Fe^3+^, can cause the oxidation of flavonoids, leading to the formation of ROS, which can, in turn, damage DNA [32]. When leukocytes were simultaneously incubated with onion extracts and H_2_O_2_, a significant decrease in DNA damage was observed (*p* < 0.0001 for *A. × cornutum* and *A. cepa*).

Hydrogen peroxide generates hydroxyl radicals (OH^●^) that cause DNA strand breaks and fragmentations. Thanks to the high levels of phenolic compounds, especially quercetin and its derivatives, *A. × cornutum* and *A. cepa* are able to catch and inactivate those radicals before they can induce any DNA damage (Figure 3). Quercetin and its glycosides may function as a donor and contribute a hydrogen atom from their phenolic hydroxyl group in the B-ring in order to remove hydroxyl radicals generated from hydrogen peroxide. Similar protective effects of quercetin on hydrogen peroxide–induced DNA damage were reported before [33,34,35]. Our results demonstrate that both extracts are able to prevent DNA damage caused by the oxidative DNA-damaging agents, such as H_2_O_2_ in human leukocytes.

### 2.5. Cell Proliferation Assay

To evaluate whether *A. × cornutum* and *A. cepa* methanolic extracts (c = 100 μg/mL) have a cytotoxic effect on breast and glioblastoma cancer cell lines, cells were treated with onion extracts for 4, 24, 48, and 72 h, and the amount of metabolically active cells was measured by MTT (3-(4,5-dimethylthiazol-2-yl)-2,5-diphenyl tetrazolium bromide) assay.

**Figure 4:**
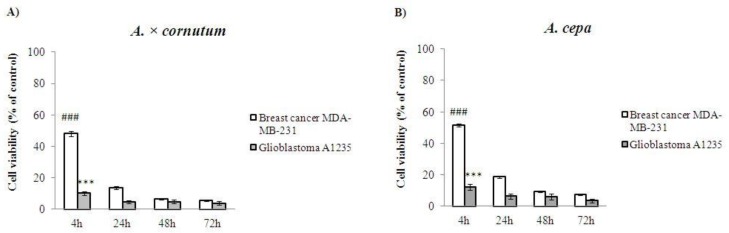
Cytotoxic effect of *A. × cornutum* and *A. cepa* methanolic extracts at concentration of 100 μg/mL on two human cancer cell lines: breast and glioblastoma cancer cells. The antiproliferative effect of *A. × cornutum* (**A**) and *A. cepa* (**B**) after 4, 24, 48 and 72 h of exposure. The percentage of metabolically active cells is expressed in comparison to the untreated control. The results are presented as a mean value ± SD of the three independent experiments. The statistically significant difference of glioblastoma cell line is represented as *** *p* < 0.001 for *A. × cornutum* and *A. cepa*, and the statistically significant difference of breast cancer cell line is represented as ### *p* < 0.001 for *A. × cornutum* and *A. cepa*.

As shown in Figure 4, both extracts significantly reduced (*** *p* < 0.001) the number of viable cells, albeit *A. × cornutum* showed a slightly stronger inhibitory effect on all cancer cell lines. The most striking observation was the low viability of glioblastoma which, among other tested cancer cells, showed the highest susceptibility in the presence of both onion extracts. Our results indicate that both plants, *A. × cornutum* and *A. cepa*, are effective inhibitors of tumor cell proliferation, which correlates with the study of Boivin et al. [26], who have also shown a strong antiproliferative effect of *Allium* vegetables on different cancer cell lines.

It is worth noting that the *Allium* extracts used in their study also displayed strong inhibitory effects against glioblastoma cancer cells. Most importantly, the authors showed that onion extract had no impact on normal fibroblast growth, suggesting that their selective antiproliferative activity was exclusively against tumor cells. Similar results were obtained by Yang et al. [36] as they confirmed the antiproliferative effect on human epithelial colorectal adenocarcinoma cells (Caco-2) and liver hepatocellular carcinoma cells (HepG2). The authors also observed that a different range of antiproliferative activities depended on the onion cultivar and the type of cancer cells used in the experimental process. Therefore, the authors hypothesized that the reason for these differences could be attributed to the different types and concentrations of bioactive phytochemicals which can target different molecules or different signaling pathways in distinct cancer cell lines. Millet et al. [37] tested the antiproliferative activity of onion extracts in different solvents: fermented aqueous extract (FAE), aqueous extract (AE), and methanolic extract (ME). Only the FAE extract showed significant toxicity to HepG2 cancer cell growth, while the other two showed no impact on cell proliferation. It should also be emphasized that the inhibition of cancer cell proliferation by the two tested extracts may not be exclusively due to their polyphenolic content, but may also be attributed to their other bioactive compounds, such as organosulfur compounds, typical in the majority of *Allium* species. These findings are in agreement with all known anticancer properties of *Allium* species noticed in many other epidemiological studies [9].

## 3. Materials and Methods

### 3.1. Chemical Reagents

Low melting point (LMP), normal melting point (NMP) agarose, hydrogen peroxide (H_2_O_2_), ethyl acetate, ethanol, methanol, ferric chloride (FeCl_3_), caffeic acid, phosphate-buffered saline (pH 7.4), dimethyl slulfoxide (DMSO), ascorbic acid, hydrochloric acid (HCl, 37%), sodium chloride (NaCl), sodium hydroxide (NaOH), sodium lauryl sarcosine, disodium salt ethylene-diamin-tetra-acetic acid (Na_2_EDTA), Trizma base, Triton X-100, Folin-Ciocalteu reagent, 2,2-diphenyl-picrylhydrazyl (DPPH), 6-hydrohy-2,5,7,8-tetramethyl-2-carboxylic acid (Trolox), fluoroshield with 4′,6-diamino-2-phenylindole (DAPI), quercetin 4′-monoglucoside, quercetin, isorhamnetin and myricetin were obtained from Sigma-Aldrich (St. Louis, MO, USA). pUC19 plasmid DNA was purchased from Invitrogen Life Technologies (Carlsbad, CA, USA). Quercetin 3,4′-diglucoside was obtained from Polyphenols AS (Sandnes, Norway), and malvidin-3-*O*-glucoside chloride was obtained from Extrasynthese (Genay, France). All chemicals and reagents were AR or HPLC grade.

### 3.2. Plant Material

*Allium × cornutum* was obtained from local gardens along the Croatian coast and islands, while the *Allium cepa* plants were purchased at a local market.

### 3.3. Preparation of Onion Extracts

The phenolic compounds were extracted from homogenized dry plant material (10 g) using 70% methanol water (*v*/*v*; 100 mL). After 30 min of extraction with magnetic stirring at room temperature (ca. 20 °C), the extract was centrifuged at 3000 rpm for 15 min. All three supernatants were pooled and dried under vacuum using a rotary evaporator (ca. 50 °C). After evaporating solvent at rotavap, samples were dried in a vacuum oven until they reached constant weight. The dry residues were dissolved with 10% DMSO. For HPLC analysis, the solution was filtered through a 0.45 µm nylon filter disc prior to analysis.

### 3.4. Analysis of the Phytochemicals

#### 3.4.1. HPLC Analysis of Flavonols

High-performance liquid chromatography (HPLC) measurement of flavonols were carried out using the Perkin Elmer HPLC system (Waltham, Massachusetts, USA) consisting of a binary pump Series 200, an autosampler, Peltier column oven Series 200, UV-VIS detector Series 200, and UltraAqueous C18 column (250 × 4.6 mm, Resek, Bellefonte, PA, USA). TotalChrom Workstation software (version 6.2.1, Perkin Elmer), was used to process the chromatographic data. After filtration through a 0.45 µm syringe filter, the extract was injected directly through a 20 µL fixed loop into a guard of the C18 column. Each sample was injected three times in order to check its reproducibility. A gradient consisting of solvent A (0.2% H_3_PO_4_) and solvent B (MeOH/acetonitrile, 1:1 *v*/*v*) was applied at a flow rate of 0.8 mL/min as follows: 0–0.5 min 96% A and 4% B; 0.5–40 min 50% A and 50% B; 40–45 min 40% A and 60% B; 45–60 min 0% A and 100% B; 60–68 min 0% A and 100% B; 68–70 min 96% A and 4% B; 70–80 min 96% A and 4% B. Detection of the elution peaks was at λ = 360 nm. Flavonoid compounds were identified on the basis of their retention times and quantified using external standard calibration curves. Standards for identification purposes were: quercetin, quercetin 4′-monoglucoside, and quercetin 3,4′-diglucoside prepared in methanol. The resultant concentrations are expressed as mg/100 g of dry weight.

#### 3.4.2. HPLC Analysis of Anthocyanins

HPLC analysis of anthocyanins was performed using a Varian HPLC system (Varian, Inc., Harbour City, CA, USA), consisting of a Star 9010 pump, a Rheodyne 7125 syringe loading sample injector, a 500-LC module for a column oven, a ProStar 330 photodiode array detector, and a Star Chromatography workstation, version 5. The separation was carried out using a Kinetex C18 core-shell column (150 × 4.6 mm), filled with 5 µm particles, and furnished with the SecurityGuard ULTRA Cartridge UHPLC C18 for 4.6 mm ID column (Phenomenex, Torrance, CA, USA), both termostated at 35 °C. Two eluents were used: A was 0.3% HClO_4_ and B was MeOH. The linear gradient was as follows: from 28% B to 51% B in 42 min, than to 69% in 3 min and to 80% B in 1 min 80% B for 3 min. The time of equilibration for the column to the initial gradient was 6 min, and the injection volume was 10 µL. The flow rate was 0.6 mL/min [38]. Samples and standards were filtered before analysis through a 0.45 µm pore size membrane syringe filters. Anthocyanins were identified according to the retention times of each peak at 520 nm. Quantifications were performed using a standard curve of malvidin-3-*O*-glucoside chloride. The resultant concentrations are expressed as mg/100 g of dry weight.

#### 3.4.3. Determination of Total Phenolic Content

The total phenolic content (TPC) of *A. × cornutum* and *A. cepa* methanolic extracts was determined using Folin-Ciocalteu method described by Singleton and Rossi [39]. Briefly, the determination of TPC in tested samples was carried out using 10 mL of previously-diluted Folin-Ciocalteu reagent (1:20 *v*/*v*) and 50 µL of onion extract. After 3 min of incubation, 1 mL of saturated sodium carbonate was added after which the reaction mixture was incubated for another 60 min in the dark and the absorbance was measured at 725 nm using the Perkin Elmer UV-VIS Lambda Bio 40 spectrophotometer (Waltham, MA, USA). Caffeic acid served as the standard and results were expressed as mg of CA equivalents per g of dry weight. All measurements were carried out in triplicate and the results are expressed as mean values ± SD.

### 3.5. Antioxidant Activity

#### 3.5.1. Measurement of the DPPH Radical Scavenging Activity

Antioxidant capacity of extracts was evaluated using the DPPH method previously described by Kulišić, Dragović-Uzelac, and Miloš [40]. This method is based on the reduction of alcoholic DPPH solution in the presence of a hydrogen-donating antioxidant. An aliquot (50 µL) of the onion extract was mixed with a methanolic solution of DPPH (1 mL, 0.1 mM) and the initial absorbance at 517 nm was measured immediately using EtOH as a blank. After 60 min of incubation, the absorbance was measured again and the percentage of DPPH inhibition was calculated according to the formula by Yen and Duh [41]:

% inhibition = ((A_C_(0) − A_A_(*t*))/A_C_(0) × 100,

where A_C_(0) is the absorbance of the control at *t* = 0 min, and A_A_(*t*) is the absorbance of the antioxidant at *t* = 1 h. All measurements were performed in triplicate.

#### 3.5.2. Oxygen Radical Absorbance Capacity Assay

The assay was performed in Perkin-Elmer LS55 spectrofluorimeter, using 96-well white polystyrene microtiter plates (Porvair Sciences, Leatherhead, UK). Each reaction contained 190 µL of fluorescein (160 µM), 60 µL 2,2'-Azobis(2-methyl-propionamidine) dihydrochloride (AAPH) (150 mM), and 30 µL of plant extracts or reference standard Trolox (6.25–50 µM). All experimental solutions and samples were prepared in a phosphate buffer (0.075 mM, pH 7.0). The measurements were performed in triplicate at 37 °C using the excitation wavelength of 485 nm, and the fluorescence decay was monitored at 530 nm during the period of 60 min. The obtained fluorescence decay curves were analyzed by FL WinLab software (version 4.00.03, Perkin-Elmer) and the resultant area under the curve (AUC) of each standard or sample was acquired after the blank AUC subtraction (Figure S1). The standard curve was generated by plotting the AUC of standards with corresponding nmol of trolox. The ORAC values of onion extracts were expressed as μmol of trolox equivalents (TE) per mL of tested sample. The results were obtained from three independent experiments.

### 3.6. Evaluation of Biological Activity

#### 3.6.1. Blood Sampling and Treatment of Human Leukocytes

Blood samples were obtained from a healthy female donor (age 28, non-smoker, the first author of this paper, and the protocol was approved by the Ethics Committee of School of Medicine, University of Zagreb, code: 380-59-10106-14-55/118, 2014). Venous blood was collected into heparinized vacutainer tubes (Becton Dickeson, Plymouth, UK) under sterile conditions. Leukocytes were isolated by gradient centrifugation with a Histopaque-1077 (Sigma) at 400× *g* for 30 min and washed twice with phosphate-buffered saline (PBS). Cell viability was determined with trypan blue exclusion assay. Leukocytes (2 × 10^6^ cells/mL) were then incubated in RPMI-1640 medium (Gibco, Invitrogen, Carlsbad, CA, USA) and supplemented with *A. × cornutum* and *A. cepa* methanolic extract (100 μg/mL) for 30 min at 37 °C in a humidified atmosphere with 5% CO_2_. Cells growing in a medium supplemented with 1% DMSO were used as the negative control and those incubated with 200 μM of H_2_O_2_ (for 5 min, 4 °C) served as the positive control. After incubation, cells were centrifuged at 300× *g* for 5 min at 4 °C and washed twice with PBS. Supernatant was discarded and the pellet was placed on ice and resuspended in LMP agarose. All treatments were performed in duplicate.

#### 3.6.2. Comet Assay (Single-Cell Gel Electrophoresis)

**Figure 5:**
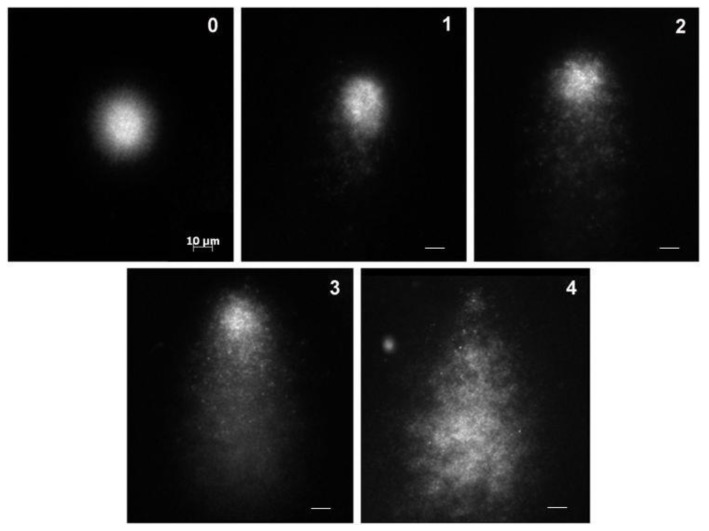
Grading of the DNA damage in human leukocytes: (**0**) no damage; (**1**) low level of DNA damage; (**2**) medium level of DNA damage; (**3**) high level of DNA damage; and (**4**) maximum level of DNA damage. Bar in the first picture (0) = 10 μm, and is also valid for all micrographs.

The alkaline comet assay was carried out according to the procedure of Singh et al. [42]. Conventional microscope slides were precoated by dipping them in a solution of 1% normal melting point agarose and left to dry overnight. After treatment, the cells were centrifuged (300× *g*, 5 min, 4 °C) and the supernatant was removed. The pellet was resuspended in the RPMI medium and the cells were counted. Approximately 1 × 10^6^ cells/mL were mixed with 100 µL 0.5% LMP agarose and placed on a precoated slide. After solidification of the agarose, the slides were immersed in fresh ice-cold lysis solution (2.5 M NaCl, 0.1 Na_2_EDTA, 10 mM Tris-Cl, 10% DMSO, 1% sodium lauryl sarcosine, 1% Triton X-100, pH 10) overnight at 4 °C. Alkaline denaturation was performed in an electrophoresis buffer solution (1 mM Na_2_EDTA and 300 mM NaOH, pH ≥ 13) for 20 min. Electrophoresis was carried out in a chilled electrophoresis buffer for 20 min (25 V, 300 mA, 4 °C). The slides were then washed three times for 5 min in 0.4 M Tris-Cl, pH 7, treated with ethanol for another 5 min, and dried. For comet analysis, the slides were stained with DAPI (5 µg/mL). A total of 100 comets (50 cells from each of the two replicated slides) were scored visually in five classes according to tail size and intensity (from undamaged 0; to maximally damaged 4) as depicted in Figure 5. DNA damage index was determined according to the equation:

DI = 1*n*1 + 2*n*2 + 3*n*3 + 4*n*4

where DI is the damage index in arbitrary units (AU), *n*1–*n*4 are the number of comets with damage levels 1, 2, 3 and 4. DI values can indicate various situations, from all undamaged cells (class 0; 0 AU) to highly damaged cells (class 4; 400 AU).

#### 3.6.3. DNA Nicking Assay

DNA nicking assay was performed using supercoiled pUC19 plasmid DNA by the method of Prakash et al. A reaction mixture containing different concentrations of plant extracts (10–100 µg/mL) and pUC19 plasmid DNA (0.5 µg) was incubated for 10 min at room temperature, followed by the addition of Fenton’s reagent (30 mM H_2_O_2_, 50 µM ascorbic acid, and 80 µM of FeCl_3_). The reaction mixture was then incubated for 30 min at 37 °C and the DNA was analyzed on a 1% agarose gel.

#### 3.6.4. Cell Culture

Cells were purchased from ATCC (LGC Standards, Bury, UK). Cancer cell lines (breast cancer cell line MDA-MB-231 and human glioblastoma cell line-A1235) were cultured in a humidified atmosphere with 5% CO_2_ at 37 °C, in a Dulbecco’s modified Eagle’s medium (DMEM Euroclone, Milano, Italy) containing 4.5 g/L glucose, 10% fetal bovine serum (FBS), and 1% antibiotics (penicillin and streptomycin, EuroClone).

#### 3.6.5. Cell Proliferation Assay

Cells were resuspended in a diluted solution of trypan blue and counted by a binocular inverted microscope, MOTIC AE30 (Motic, Barcelona, Spain), using Neubauer chambers. The cell number was calculated according to the formula: number of counted cells × 10^4^/mL. The cells were then plated in 96-well plates at a density of 11,000 cells/well and incubated overnight. The cells were treated with *A. × cornutum* and *A. cepa* methanolic extract at a concentration of 100 µg/mL in a complete medium (in triplicate) for 4, 24, 48 and 72 h. Then, the MTT assay was performed in such a manner that after the treatment with onion extracts, the cells were incubated with 0.5 g MTT/L at 37 °C for 2 h. After that, the medium was removed and dimethylsulphoxide (10% DMSO) was added and incubated for another 10 min at 37 °C while shaking. The degree of formazan formation, an indicator of living and metabolically active cells, was measured photometrically at 570 nm. The data was calculated in relation to the untreated control (100%) from three independent measurements.

### 3.7. Statistical Analysis

Results are expressed as mean values with depicted standard deviation. Microsoft Excel Student *t*-test was used to analyze data and to discriminate statistically significant results.

## 4. Conclusions

This was the first known thoroughly comprehensive study of the phytochemical composition and biological activity of *A. × cornutum*. The HPLC analysis revealed two major quercetin conjugates (quercetin 3,4′-diglucoside and quercetin 4′-monoglucoside) as the most abundant flavonols in *A. × cornutum* and *A. cepa* extracts. Additionally, we successfully identified three anthocyanins in both onions, while the fourth anthocyanin, delphinidin glucoside, was observed only in *A. × cornutum*. Overall, *A. × cornutum* showed slightly higher concentrations of all identified phenolic compounds, which may be the cause of the higher bioactivity profile accounted for in this onion.

Our results clearly indicate that both onions have strong protective effects on the DNA molecule, as was proven with several different in vitro experiments. Both onions have also shown strong antiproliferative activity on human cancer cell lines; however, the susceptibility of glioblastoma cells against both onion extracts was higher than that of a breast cancer cell line. The antiproliferative activity of the tested methanol extracts could be mediated by the induction of apoptosis, alterations of the cell cycle, or some other mechanism, while further research is needed to clarify the exact mechanism(s) of their antiproliferative activity in vitro. Therefore, it can be concluded that *A. × cornutum* and *A. cepa* methanolic extracts have a comparable composition and concentrations of phenolic compounds, as well as antioxidant, antigenotoxic and antiproliferative effects. This research demonstrated that *A. × cornutum* and common onions should be considered as important sources of natural antioxidants that have a beneficial protective effect on human health.

## Figures and Tables

**Table 1:** HPLC quantification of flavonols and anthocyanins in *A. cepa* and *A. × cornutum* extracts.

	*A. × cornutum* (*t*_R_, min)	*A. cepa* (*t*_R_, min)
**Flavonols ^a^**		
Quercetin 3,4′-diglucoside (**1**)	240.01 ± 0.39 (33.30)	171.34 ± 0.13 (33.26)
Quercetin 4′-monoglucoside (**2**)	159.86 ± 0.09 (42.45)	117.38 ± 0.17 (41.41)
Myricetin (**3**)	6.22 ± 0.09 (43.37)	8.02 ± 0.02 (43.34)
Quercetin aglycone (**4**)	24.13 ± 0.08 (48.17)	19.85 ± 0.03 (48.13)
Isorhamnetin (**5**)	7.43 ± 0.05 (52.97)	4.74 ± 0.01 (52.94)
**Anthocyanins ^a^**		
Peonidin 3′-glucoside (**6**)	0.54 ± 0.00 (11.35)	0.19 ± 0.00 (12.11)
Petunidin 3′-glucoside acetate (**7**)	0.52 ± 0.00 (21.72)	0.13 ± 0.01 (22.58)
Delphinidin 3′-glucoside (**9**)	0.15 ± 0.00 (31.37)	Nd
Malvidin 3′-glucoside (**8**)	0.01 ± 0.00 (39.27)	0.03 ± 0.00 (38.87)

^a^ Concentrations in mg/100 g of dry weight; *t*_R_, retention time.

**Table 2:** Antioxidative potential of *A. × cornutum* and *A. cepa* methanolic extracts determined by DPPH and ORAC methods.

Antioxidant Assay	*A. × cornutum* (100 μg/mL)	*A. cepa* (100 μg/mL)
DPPH (% DPPH inhibition)	60.50 ± 3.84	64.82 ± 5.31
ORAC (µmol TE/mL)	19.38 ± 2.21	17.62 ± 0.57

DPPH, 2,2′-diphenyl-1-picrylhydrazyl; ORAC, oxygen radical absorbance capacity.

## Supplementary Materials

Supplementary materials are available online. Figure S1. Fluorescence decay curve of methanolic extracts of *A. cepa* (- - -), *A. × cornutum* (---) and blank (―); Figure S2. High-performance liquid chromatography (HPLC) chromatogram of *A. × cornutum* anthocyanins at 520 nm. Depicted are peaks: peonidine 3'-glucoside, petunidin 3'-glucoside, delphidin 3'-glucoside and malvidin 3'-glucoside; Figure S3. High-performance liquid chromatography chromatogram of *A. cepa* anthocyanins at 520 nm. Depicted are peaks: peonidine 3'-glucoside, petunidin 3'-glucoside and malvidin 3'-glucoside.
