# Supplementary material for: Chemical Composition and Biological Activity of Allium cepa L. and Allium × cornutum (Clementi ex Visiani 1842) Methanolic Extracts
Source: Molecules. 2017 Mar 11;22(3):448. doi: 10.3390/molecules22030448 (PMC6155300; doi:10.3390/molecules22030448)
Supplement: Supplementary file 1 [file molecules-22-00448-s001.pdf]

Chemical Composition and Biological Activity of *Allium cepa* L. and *Allium* × *cornutum* (Clementi ex Visiani 1842) Methanolic Extracts

Željana Fredotović, Matilda Šprung, Barbara Soldo, Ivica Ljubenkov, Irena Budić-Leto, Tea Bilušić, Vedrana Čikeš-Čulić and Jasna Puizina

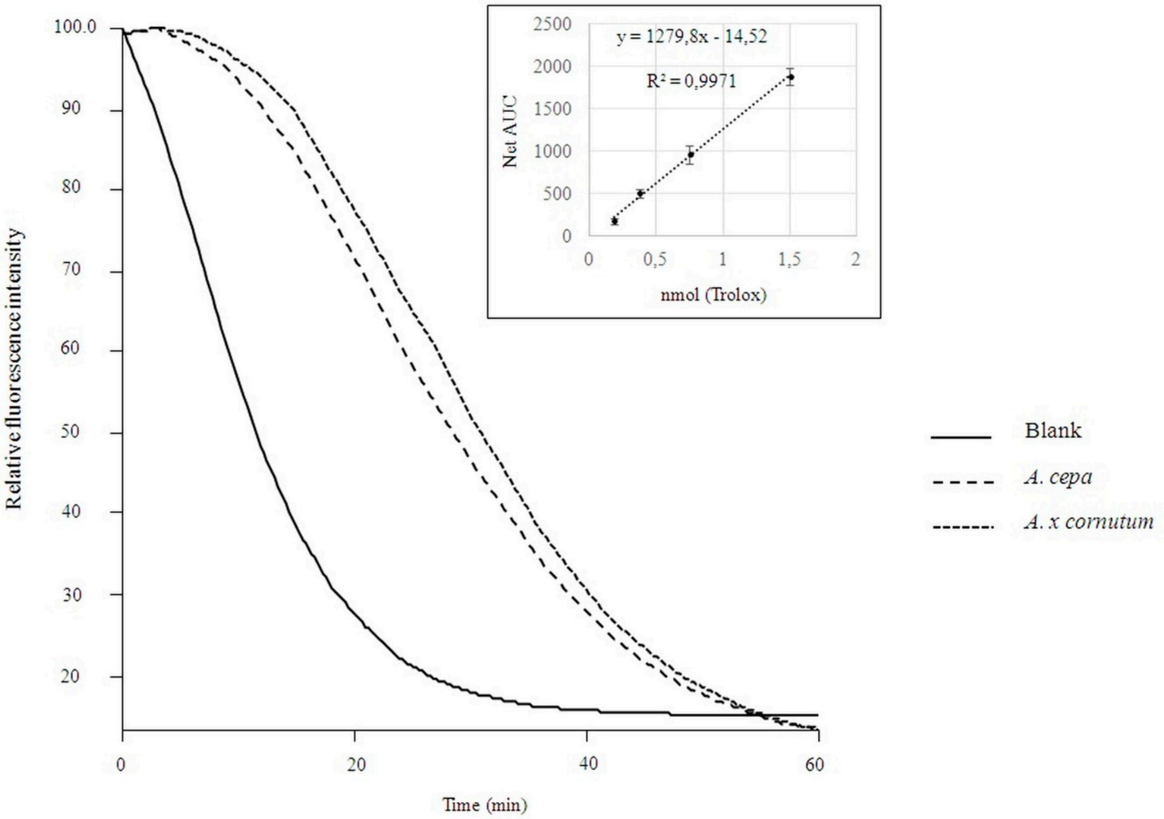

Figure S1. Fluorescence decay curve of methanolic extracts of *A. cepa* (- - -), *A.* × *cornutum* (---) and blank (—).

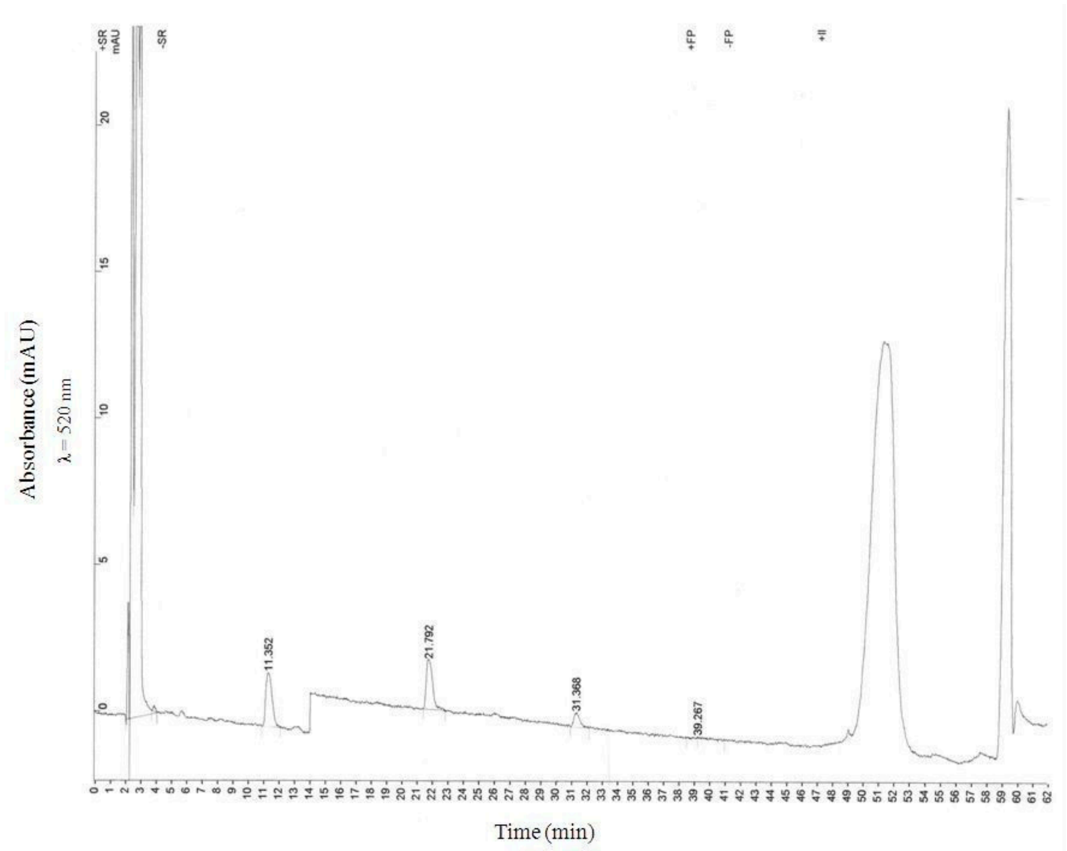

Figure S2. High-performance liquid chromatography (HPLC) chromatogram of *A.* × *cornutum* anthocyanins at 520 nm. Depicted are peaks: peonidine 3'-glucoside, petunidin 3'-glucoside, delphidin 3'-glucoside and malvidin 3'-glucoside.

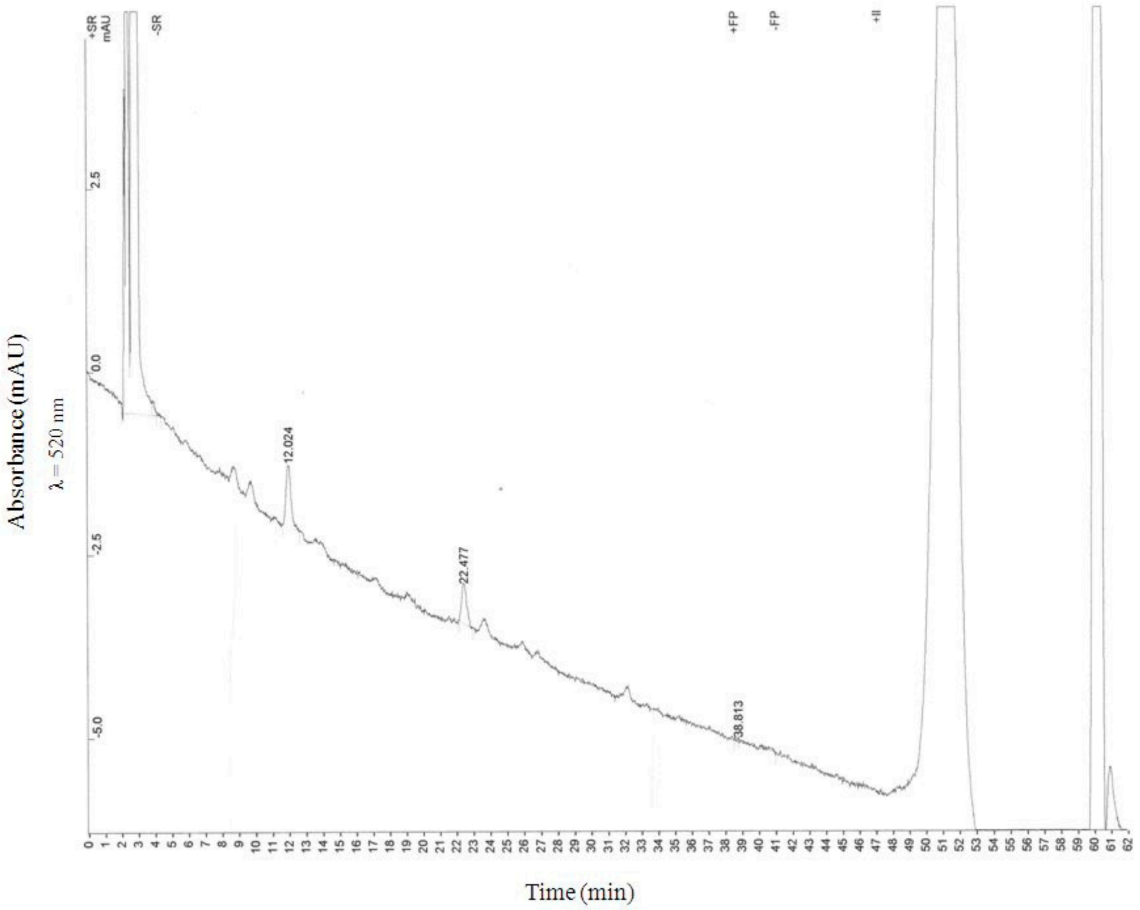

Figure S3. High-performance liquid chromatography chromatogram of *A. cepa* anthocyanins at 520 nm. Depicted are peaks: peonidine 3'-glucoside, petunidin 3'-glucoside and malvidin 3'-glucoside.
